# A temperate river estuary is a sink for methanotrophs adapted to extremes of pH, temperature and salinity

**DOI:** 10.1111/1758-2229.12359

**Published:** 2016-01-22

**Authors:** Angela Sherry, Kate A. Osborne, Frances R. Sidgwick, Neil D. Gray, Helen M. Talbot

**Affiliations:** ^1^ School of Civil Engineering and GeosciencesNewcastle UniversityNewcastle upon TyneNE1 7RUUK

## Abstract

River Tyne (UK) estuarine sediments harbour a genetically and functionally diverse community of methane‐oxidizing bacteria (methanotrophs), the composition and activity of which were directly influenced by imposed environmental conditions (pH, salinity, temperature) that extended far beyond those found *in situ*. In aerobic sediment slurries methane oxidation rates were monitored together with the diversity of a functional gene marker for methanotrophs (*pmoA*). Under near *in situ* conditions (4–30°C, pH 6–8, 1–15 g l^−1^ NaCl), communities were enriched by sequences affiliated with *M* *ethylobacter* and *M* *ethylomonas* spp. and specifically a *M* *ethylobacter psychrophilus*‐related species at 4–21°C. More extreme conditions, namely high temperatures ≥ 40°C, high ≥ 9 and low ≤ 5 pH, and high salinities ≥ 35 g l^−1^ selected for putative thermophiles (*M* *ethylocaldum*), acidophiles (*M* *ethylosoma*) and haloalkaliphiles (*M* *ethylomicrobium*). The presence of these extreme methanotrophs (unlikely to be part of the active community *in situ*) indicates passive dispersal from surrounding environments into the estuary.

## Introduction

Methanotrophs (methane‐oxidizing bacteria) are nature's only biological mechanism for suppressing methane release to the atmosphere from anaerobic sediments/soils (Hanson and Hanson, 1996; Nazaries *et al*., 2013; Gray *et al*., 2014). This process is important because methane accounts for up to 20–30% of the global warming effect (IPCC, 2007). Methanotrophs and methane oxidation have been studied extensively in terrestrial environments such as soils (Martineau *et al*., 2010; 2014), including landfill cover (Su *et al*., 2014), wetlands (Danilova and Dedysh, 2014), peat bogs (Kip *et al*., 2010; Putkinen *et al*., 2014) and in freshwater aquatic systems (Blees *et al*., 2014; Zigah *et al*., 2015). By contrast, aerobic methane oxidation in marine and estuarine systems has received little attention (Holmes *et al*., 1995; Valentine, 2011) despite the obvious importance of methanotrophy in limiting methane release to the atmosphere as observed following the blowout of the Macondo oil well in the gulf of Mexico (Crespo‐Medina *et al*., 2014) and the finding that estuaries play an important role in methane cycling (Abril and Iversen, 2002; McDonald *et al*., 2005).

Recent studies have used estuaries as settings in which to study the influence of environmental parameters on microbial diversity *in situ*, for example, sulfate‐reducing bacteria (Purdy *et al*., 2002), methanogens (Oakley *et al*., 2012), and ammonia‐oxidizing bacteria and archaea (Mosier and Francis, 2008). The rationale for this current study is that estuaries offer a dynamic but constrained link between freshwater/terrestrial and marine environments, and likely act as sinks for particulate material from these sources. For instance, the River Tyne estuary on the north‐east coast of England receives inputs of 350 000 tonnes of freshwater and 250 000 tonnes of marine sediment per year (Hall, 1967). Additionally, turbidity maxima of estuaries can retain particulate material for extended periods of time (Goosen *et al*., 1999). Furthermore, tidal processes give rise to large variations in salinity, and for the Tyne acidic run‐offs occur from peat and acid mine drainage, and there are also inputs derived from industrial, agriculture and composting activities (Upstill‐Goddard *et al*., 2000; Baker and Spencer, 2004; Starkey *et al*., 2015).

We investigated methane oxidation and methanotroph diversity in sediments from the River Tyne estuary, UK, to explore the environmental limits of the *in situ* ‘adapted’ community and to identify methanotrophs present but adapted to more extreme conditions. We systematically assessed the response of sedimentary methanotroph communities to variations in temperature, methane concentration, pH and salinity in a single sampling location using aerobic sediment slurry incubations.

## Results and discussion

In all of the sediment slurry experiments (Table S1), incubations were characterized by two phases of methanotroph activity, namely an initial lag phase without methane consumption and a period of methane removal, which was linear and from which maximal methane oxidation rates were calculated. In this study, formal statistical comparisons of methane oxidation rates (analysis of variance with Tukey's honestly significant difference) were only made between treatments and controls that tested a single condition, e.g. temperature, since the different experiments were not run concurrently and were prepared with different batches of sediment and freshly prepared enrichment media.

**Figure 1:**
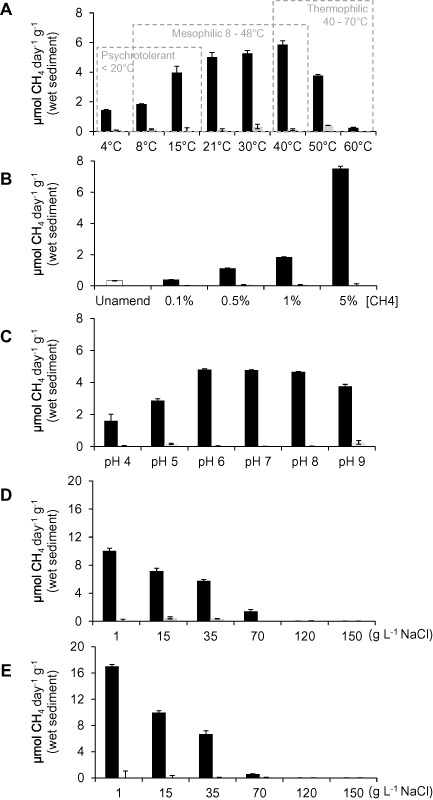
Methane oxidation rate in response to variations in (A) temperature, (B) CH_4_ concentration, (C) pH, (D) salinity at 21°C and (E) salinity at 40°C in sediment slurries from the River Tyne estuary. Methane‐amended incubations (black bars) contained 5% CH_4_ (except where indicated in B), and heat‐killed controls (grey bars) were autoclaved prior to 5% CH_4_ addition (except where indicated in B). Error bars represent 1 × SE (*n* = 3). Sediment slurries were prepared in glass serum bottles (60 ml) that comprised sterile growth medium (22 ml, Widdel and Bak, 1992), homogenized surface sediment (∼ 3.5 g) and headspace (35 ml). Growth medium in sediment slurries was pH 7.5 ± 0.7 with 7 g l^−1^ NaCl, incubation was at 21°C with 5% CH4 addition to the headspace, except where indicated. Experimental set‐up and incubations were staggered with the different experiments (A–E) prepared with separate batches of sediment and freshly prepared growth medium.

**Figure 2:**
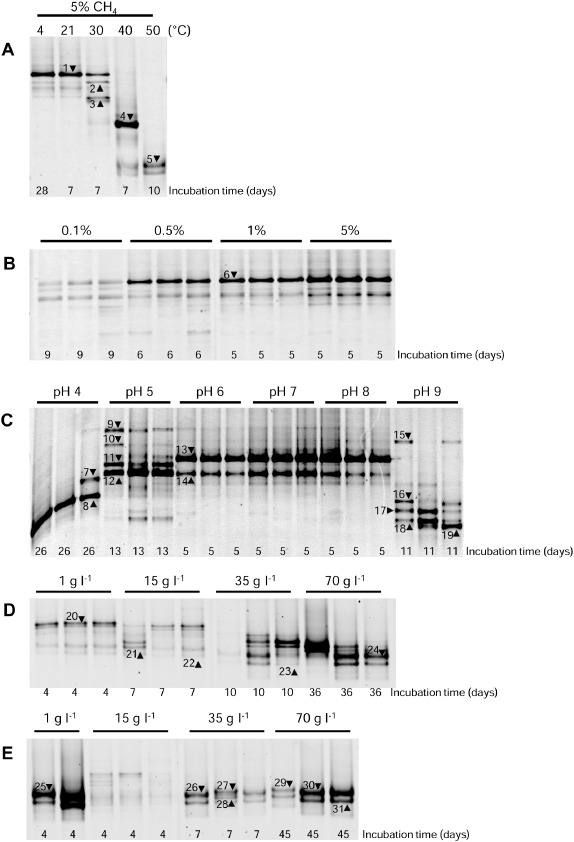
PCR‐DGGE of the *pmoA* gene as an indicator of methanotroph community composition changes in River Tyne sediment slurry incubations following enrichment under the following environmental conditions: (A) temperature, (B) CH_4_ concentration, (C) pH, (D) salinity at 21°C and (E) salinity at 40°C. The length of incubation (days) was determined from Time 0 to the removal of methane in headspace (5%, except where indicated in B), noted below each lane. It should be noted that for temperature, only one of the replicate profiles for each temperature is shown. Reproducibility for this environmental condition was assessed on a separate gel (data not shown) and was found to be highly reproducible. Differences in methanotroph diversity determined in this study are reflective of compositional changes during enrichment under different imposed environmental conditions. DNA was extracted from destructively sampled enrichment slurries after methane removal. PCR‐DGGE was used to monitor changes in methanotroph communities by targeting the particulate methane monooxygenase gene (*pmoA*), numbered bands indicate those that were excised, re‐amplified and purified for DNA sequencing.

**Figure 3:**
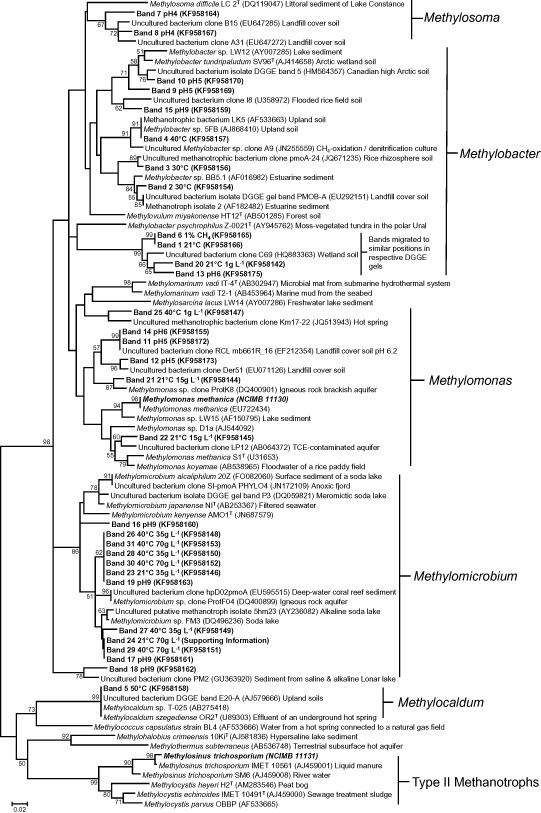
Neighbour‐joining tree based on partial *pmoA* gene nucleotide sequences. Bootstrap confidence values of ≥ 50% from 1000 replicates are indicated at branch nodes. Methanotroph *pmoA* gene sequences from Type II *A* *lphaproteobacteria* are shown as an outgroup. Methanotroph type strains are depicted by ^T^. GenBank accession numbers are shown in parentheses. Scale bar represents 0.02 substitutions per base position.

In a general assessment of the trends in methane oxidation rates (Fig. 1), which are discussed in more detail below, we found decreased rates of methane oxidation with increasing salinity and increased rates of methane oxidation with increasing methane concentration. In contrast there were clear optima for both temperature and pH variation. Superficially, these results are consistent with the growth characteristics of cultured methanotrophs. For instance, the Type I methanotroph *Methylomonas koyamae* grows over a very broad range of methane concentrations with increasing specific growth rates up to 10% methane (Ogiso *et al*., 2012). This organism also grows optimally at a temperature of 30°C with a growth range of 10–40°C and optimally at pH 6.5 with a growth range of 5.5–7. In contrast, both the Tyne methanotroph community and *Methylomonas koyamae* grow best at salinities (0–10 g l^−1^ NaCl) considerably lower than their maximum tolerances (Tyne methanotrophs ≈70 g l^−1^ NaCl and *M. koyamae* 50 g l^−1^ NaCl). However, with the exception of methane concentration, our Tyne experiments revealed a range of growth conditions broader than the growth characteristics of individual methanotroph cultures. The extremes of these ranges were accompanied by highly reproducible shifts in community composition determined in this study after complete methane removal from enrichment slurries by Polymerase Chain Reaction‐Denaturing Gradient Gel Electrophoresis (PCR‐DGGE) (targeting the particulate methane monooxygenase gene [*pmoA*] coupled to band excision and sequencing; Figs 2 and 3).

It should be noted for the subsequent results and discussion sections that in this study all excised pmoA gene sequences were affiliated with Type I methanotrophs rather than Type II methanotrophs, which include the genera *Methylocapsa*, *Methylosinus*, and *Methylocystis*. An alternative PCR assay using 16S rRNA gene primers targeting the *Methylosinus* and *Methylocystis* Type II methanotrophs also did not generate a visible PCR amplicon in any of the sample extracts as was observed for the control (*Methylosinus trichosporium* OB3b). The dominance of Type I over other methanotrophs in the incubation slurries, however, cannot be unequivocally concluded as the PCR‐DGGE approach is not comprehensive (McDonald *et al*., 2008) and certainly would not target Type II methanotrophs that lack pMMO, i.e. *Methylocella* spp. (Dedysh *et al*., 2000; Dunfield *et al*., 2003), *Methyloferula stellata* (Vorobev *et al*., 2011), or the extremely acidic methanotrophic *Verrucomicrobia* (Dunfield *et al*., 2007; Op den Camp *et al*., 2009).

### Identification of psychrotolerant, mesophilic and thermophilic methanotrophs in the River Tyne estuary

Tyne methanotrophs were active over a broad range of temperatures from 4°C to 50°C, with rates of maximal methane oxidation in all these methane‐amended slurries significantly higher than rates in corresponding heat‐killed methane‐amended controls (*P* < 0.001 for 4–50°C, Fig. 1A). Rates at 60°C showed no significant difference to heat‐killed controls (*P* = 0.898). At the lowest temperatures (4°C and 8°C), the microbial lag phases before the onset of maximal methane oxidation were 13 and 10 days, respectively, compared with the shorter lag phases of 3 days over the temperature range 15–40°C and 2 days at 50°C (Table S1). Trotsenko and Khmelenina (2005) showed that *in vivo* methane oxidation rates as well as the *in vitro* activities of enzymes decrease substantially between 30°C and 5°C in psychrophilic‐psychrotolerant methanotrophs. These results, therefore, suggest that, in addition to mesophiles, psychrotolerant methanotrophs are also present in the River Tyne and are active at low‐ambient temperatures (< 4–20°C, Fig. 1A). Further, thermophilic methanotrophs were active at moderately high temperatures (40–50°C) albeit not at the upper temperature limit (67°C) found for the most thermophilic methanotroph *Methylothermus thermalis* (Tsubota *et al*., 2005).

Methanotroph diversity in slurries incubated at 4°C and 21°C showed the same DGGE profiles comprising one dominant band (Band 1, Fig. 2A), which in support of the presence of psychrotolerant methanotrophs in the Tyne was most closely related (96% sequence identity) with an environmental clone (C69) of an uncultured *Methylobacter* sp. from a wetland soil on the cold Zoige Tibetan plateau (HQ883363, Table S2, Fig. 3). The closest cultured representative was *Methylobacter psychrophilus* isolated from a moss‐vegetated area on the tundra in the polar Ural (AY945762, Fig. 3). This band was also present at 30°C, although less dominant (Fig. 2A). In contrast, Band 2 at 30°C (also present but fainter at the lower temperatures) was most closely related to *Methylobacter* sp. BB5.1 (AF016982, Fig. 3) and a methanotroph isolate 2 (93%, AF182482, Table S2) isolated from estuarine sediments in Southern California, where such *pmoA* sequences dominated, albeit with *Methylomicrobium* and Type II methanotrophs *Methylosinus* (McDonald *et al*., 2005). Band 3, which was also dominant at 30°C (but also present but fainter at lower temperatures, Fig. 2A), was closely related to an uncultured *Methylobacter* related bacterium (clone pmoA‐24) from a rice rhizosphere soil (97%, Fig. 3 and Table S2).

At 40°C, Bands 1–3 were absent, indicating a transition in the community in response to increasing temperature (Fig. 2A). At this temperature, Band 4 shared 100% sequence identity with the methanotrophic bacterium LK5 (AF533663) isolated from an upland soil and 97% sequence identity with an uncultured *Methylobacter* sp. clone A9 (JN255559) (Fig. 3, Table S2) identified in a laboratory reactor treating landfill leachate. At 50°C, Band 4 was itself absent and the dominant band (Band 5, Fig. 2A) shared 100% sequence identity to the cultured methanotroph *Methylocaldum* sp. T‐025 (AB275418) and *Methylocaldum szegediensis* OR2 (U89303, Fig. 3, Table S2). *Methylcaldum* spp. are putatively thermotolerant or thermophilic methanotrophs (Bodrossy *et al*., 1997; Dunfield, 2009), i.e. *Methylocaldum szegediensis* OR2^T^ isolated from an underground hot spring with optimum growth at 55°C (Bodrossy *et al*., 1995). However, it is interesting to note that *Methylcaldum* spp. and other putative thermophilic methanotrophs have been identified in, or even isolated from, relatively cold environments such as soils (Bodrossy *et al*., 1997; Knief *et al*., 2003). From the systematic approach taken in this study, we can now more precisely relate the presence/absence and activity of such thermophiles to their mesophilic and psychrotolerant counterparts as has been previously determined for methanogens in rice paddy and arctic soils (Fey and Conrad, 2000; Blake *et al*., 2015). Given the cold *in situ* Tyne estuary conditions, it can be concluded that such thermophiles must have been passively dispersed in a viable state from hot extra‐estuarine sources; however, the short lag phase observed (2 days, Table S1) suggests rapid cell growth and/or rapid enzymatic and metabolic adaptations to increases in temperature (Trotsenko *et al*., 2009). Possible but as yet untested sources for thermophiles in the Tyne river catchment are likely to be large‐scale composting facilities or industrial cooling waters.

### Methanotroph function and diversity in response to methane concentration

Methane oxidation rates in methane‐amended slurries were significantly higher than in the corresponding heat‐killed controls (*P* ≤ 0.024 across all CH_4_ concentrations). The background level of methane in the un‐amended slurries was 5.46 ± 0.22 μmol CH_4_, which was oxidized at 0.33 μmol CH_4_ day^−1^ g^−1^ wet sediment (Fig. 1B, unamended). Oxidation rates in response to all methane amendments (except 0.1% methane, *P* = 0.997) were progressively and significantly higher than those in the unamended slurries (*P* < 0.001, Fig. 1B). This response to increasing methane concentration was apparently linear over the range of methane concentrations tested, with saturation for methane not reached at 5% CH_4_. The microbial lag phase before the onset of methane oxidation was relatively long in sediment slurries subjected to low methane concentrations (0.1%), compared with those amended with 0.5, 1 or 5% methane, which showed relatively short lag phases of 2 days (Table S1). The PCR‐DGGE profiles of the *pmoA* gene across all methane concentrations were, however, very similar to each other regardless of methane concentration (Fig. 2B) and were apparently similar to the lower temperature profiles with the dominant band (Band 6, Figs 2B and 3, Table S3) also related to an uncultured *Methylobacter* sp. from a wetland soil on the cold Zoige Tibetan plateau as found for Band 1.

### Identification of methanotrophs at moderate pH and low salinities in the River Tyne estuary

Rates of methane oxidation in all the pH‐adjusted methane‐amended sediment slurries were significantly higher than in corresponding heat‐killed controls (*P* < 0.001 for pH 4–9, Fig. 1C). However, at pH values similar to those *in situ* (pH 7.99), there was also no significant difference between methane oxidation rates at pH values of 6, 7 and 8 (*P* = 1.0, Fig. 1C), consistent with little observed variation in methanotroph community composition over the same conditions (pH 6–8, Fig. 2C). Critically, these profiles resembled those also observed in the low temperature and all the methane concentration experiments. The dominant band (Band 13) was related to Bands 1 and 6 identified in these other experiments (Fig. 2A and B, Fig. 3), for these pH values the microbial lag phase before the onset of methane oxidation was short (1 day, Table S1).

With respect to salinity, there were significant differences in the rates of oxidation in methane‐amended sediment slurries compared with heat‐killed slurries at 1, 15 and 35 g l^−1^ NaCl at both temperatures tested, namely 21°C and 40°C (*P* < 0.001, Fig. 1D and E), but not at 70, 120 and 150 g l^−1^ NaCl (*P* = 1.0, except 70 g l^−1^ at 21°C [*P* = 0.051]; Fig. 1D and E). However, at the lowest salinity tested (1 g l^−1^ NaCl) at both 21°C and 40°C, the lag phases before the onset of methane oxidation were short (2 days, Table S1 and likewise for incubations containing 15 g l^−1^ NaCl at 40°C, compared with the slightly longer 3.8 day lag phase at 21°C; Table S1). The DGGE profiles for these low salinity enrichments (Fig. 2C) were again similar to those described above with Band 20 found to be closely related to the *Methylobacter* related Bands 1, 6 and 13 (Fig. 3). Band 21, also present in the low temperature sediment slurries and all methane concentration incubations, was most closely related to the *Methylomonas* sp. clone ProtK8 (98% sequence identity, DQ400901) from an igneous rock brackish aquifer (Table S5).

### Identification of halo‐ and alkalitolerant methanotrophs in the River Tyne estuary

To date several halophilic/halotolerant and haloalkaliphilic/haloalkalitolerant methanotrophic species have been isolated (Khmelenina *et al*., 1997; Kalyuzhnaya *et al*., 1998; 1999; 2001; 2008; Sorokin *et al*., 2000). *Methylohalobius crimeensis* isolated from hypersaline lakes grows optimally up to 87 g l^−1^, with growth up to 150 g l^−1^ NaCl (Heyer *et al*., 2005), and there are reports of methanotroph activities up to 300 g l^−1^ NaCl (Dunfield, 2009). Further, a *Methylomicrobium* sp. has been shown to oxidize methane optimally at pH 10, with cell activity still present at pH 11 (Sorokin *et al*., 2000). In the Tyne, methane oxidation rates decreased with increasing salinity at both temperatures (Fig. 1D and E). At the highest salinity with measurable methane oxidation (70 g l^−1^ NaCl), lag phases had increased to 17 days at 21°C and 11 days at 40°C (Table S1). To a lesser extent methane oxidation rates were also significantly lower when the sediment pH was raised to 9 (*P* < 0.001, Fig. 1C), and lag phases were increased to 6 days (Table S1). Critically, both of these salinity and pH conditions selected for methanotroph sequences, which are putatively halo/alkalitolerant methanotrophs. At pH 9 although a *Methylobacter* spp. was present (Band 15), the slurries were mostly dominated by *Methylomicrobium spp.* (Bands 16–19, Fig. 2C, Table S4, Fig. 3). The closest cultured representatives included *Methylomicrobium alcaliphilum* str. 20Z and *Methylomicrobium kenyense* strain AMO1, and uncultured environmental representatives from hypersaline and alkaline lakes, anoxic fjords, and the marine environment (Fig. 3, Table S4). Sediment slurries containing 35 g l^−1^ NaCl and incubated at 21°C (Band 23, Fig. 2D) and 40°C (Bands 26 and 28, Fig. 2E), and those containing 70 g l^−1^ NaCl at 40°C (Bands 30 and 31, Fig. 2E), were also dominated by *Methylomicrobium* spp. most closely related to *Methylomicrobium japanense* NI (96–97% sequence identity, AB253367, Table S5) isolated from filtered seawater. The most closely related environmental *pmoA* sequence was *Methylomicrobium* sp. clone ProtF04 (96–97% sequence identity, DQ400899, Table S5) identified from a study of methanotrophs in igneous rock aquifers. At 35 g l^−1^ NaCl and 70 g l^−1^ NaCl at 40°C, Bands 27 and 29 (Fig. 2E) were related to *Methylomicrobium alcaliphilum* str. 20Z (96–97% sequence identity, FO082060, Table S5, Fig. 3).

This selection for different methanotrophs clearly not adapted to *in situ* conditions, especially at the higher salinities, is intriguing given that none of the sequences identified were affiliated with known marine methanotrophs, such as *Methylomarinum spp*. isolated from shallow submarine hydrothermal systems in Japan (Hirayama *et al*., 2007, Hirayama *et al*., 2013, Hirayama *et al*., 2014). It has been previously speculated by Munson and colleagues (1997) that exposed mudflats or salt marshes in estuaries are subject to drying out where salinities can be high and consistent with the presence of halophilic archaea. Such mudflats are certainly a key feature of the tidal reaches of the Tyne and may explain the presence of these apparently salinity tolerant *Methylomicrobium* spp.

### Identification of acidophilic methanotrophs in the River Tyne estuary

To an even greater extent than for the high pH enrichments, lower methane oxidation rates were also observed at the other end of the scale, namely at pH 4 and 5 (*P* < 0.001, Fig. 1C). For these enrichments the lag phases were extended up to 13 days (Table S1) and selected for different methanotroph sequences, which in this case are putative acidophiles. Acidophilic methanotrophs include species related to the Alphaproteobacterial genera *Methylocella*, *Methylocapsa*, *Methylocystis* and *Methylosinus* (Dedysh *et al*., 1998; 2000; 2002; Dunfield *et al*., 2003; Kip *et al*., 2011), and extremely acidic methanotrophic *Verrucomicrobia* (Dunfield *et al*., 2007; Op den Camp *et al*., 2009). However, no enrichment of *Methylocapsa*, *Methylocystis* or *Methylosinus* spp. was observed in the acidic Tyne sediment slurries. In the case of *Methylocella* spp. and the methanotrophic *Verrucomicrobia*, none of the PCR assays used in this study would have identified their enrichment, and so their presence or absence remains to be determined. Nevertheless, acidophilic Gammaproteobacterial methanotrophs are now known (Kip *et al*., 2011), including *Methylomonas* spp. and a putatively novel clade related to the genera *Methylosoma*/*Methylovulum*, which is of interest because of the strong enrichment of related organisms in sediment slurry incubations at pH 4 and 5 (Figs 2 and 3). For instance, Band 11 at pH 5 shared 98% sequence identity to an uncultured bacterium clone RCL_mb661R_16 (EF212354) isolated from a landfill cover soil. The closest cultured representative was *Methylomonas methanica* (92%, EU722434, Table S4, Fig. 3). Band 12 was also a *Methylomonas* sp. with 98% sequence identity with an uncultured bacterium clone Der51 (EU071126) from a landfill cover soil (Figs 2C and 3, Table S4). Bands 7 and 8 from pH 4 slurries were related to *Methylosoma* spp., and in particular Band 8 was strongly selected but shared only 92% sequence identity to an uncultured bacterium clone B15 (EU647285, Fig. 3, Table S4) isolated from a landfill cover soil. The closest cultured representative was *Methylosoma difficile LC 2* ***^T^*** (DQ119047) isolated from the littoral sediment of Lake Constance (Fig. 3). Clearly, such examples of putative acidophilic methanotrophs are unlikely to be part of the indigenous active populations within Tyne estuarine sediments. Presumably, these microorganisms with low abundance as indicated by long lag phases have been passively dispersed into the sediments as also suggested for the thermophilic *Methylocaldum*; however, for these acidophiles, an obvious source would be acidic peat soils within the upper Tyne catchment at Haltwhistle, and/or from agricultural run‐off from the Kielder Reservoir in Northumberland National Park during weekly releases.

## Conclusion

This study has determined the environmental limits of methanotrophs that can be considered indigenous and active within the Tyne sediment but has also revealed the presence of likely inactive, but viable methanotrophs, able only to respond to more extreme imposed conditions. Previously, it has been suggested that formation of resistant cells (cysts and exospores) enables methanotrophs to persist for long periods under harsh conditions (Whittenbury *et al*., 1970), and that this property probably allows for succession in methanotroph communities (Krause *et al*., 2010), with resting stages thought to provide a seed bank from which methanotrophs can periodically become active and contribute to and maintain methane oxidation as conditions vary on a seasonal basis (Eller *et al*., 2005). In the current study we have shown that temperature variation in the River Tyne may favour such a dynamic coexistence, i.e. psychrotolerant and mesophilic methanotrophs; however, the acidophiles, haloalkalophiles and thermophiles identified indicate a different biological process in action, namely passive dispersal. These dispersed methanotrophs are never likely to contribute to a seed bank for maintaining function unless they continue on their journey and are ultimately reintroduced to an environment similar to that from which they came.

## Supporting information


**Table S1.** Environmental conditions and lag phase (days) before the onset of methane oxidation in aerobic River Tyne sediment slurry incubations.
**Table S2.** Sequence identity of methanotrophs in aerobic methane‐oxidizing sediment slurry incubations incubated at different temperatures.
**Table S3.** Sequence identity of methanotrophs in aerobic methane‐oxidizing sediment slurry incubations in response to methane concentration.
**Table S4.** Sequence identity of methanotrophs in aerobic methane‐oxidizing sediment slurry incubations in response to pH.
**Table S5.** Sequence identity of methanotrophs in aerobic methane‐oxidizing sediment slurry incubations in response to salinity.
**Appendix S1.** Experimental procedures.
