# Supplementary material for: A temperate river estuary is a sink for methanotrophs adapted to extremes of pH, temperature and salinity
Source: Environ Microbiol Rep. 2016 Jan 22;8(1):122–31. doi: 10.1111/1758-2229.12359 (PMC4959530; doi:10.1111/1758-2229.12359)
Supplement: Supplementary file 1 — Table S1. Environmental conditions and lag phase (days) before the onset of methane oxidation in aerobic River Tyne sediment slurry incubations. Table S2. Sequence identity of methanotrophs in aerobic methane‐oxidizing sediment slurry incubations incubated at different temperatures. Table S3. Sequence identity of methanotrophs in aerobic methane‐oxidizing sediment slurry incubations in response to methane concentration. Table S4. Sequence identity of methanotrophs in aerobic methane‐oxidizing sediment slurry incubations in response to pH. Table S5. Sequence identity of methanotrophs in aerobic methane‐oxidizing sediment slurry incubations in response to salinity. Appendix S1. Experimental procedures. [file EMI4-8-122-s001.docx]

Supporting Information

A temperate river estuary is a sink for methanotrophs adapted to extremes of pH, temperature and salinity.

Angela Sherry,^*^ Kate A. Osborne, Frances R. Sidgwick, Neil D. Gray and Helen M. Talbot

School of Civil Engineering & Geosciences, Newcastle University, Newcastle upon Tyne, NE1 7RU, UK.

^*^For correspondence. Email [angela.sherry@ncl.ac.uk](mailto:angela.sherry@ncl.ac.uk); Tel. (+44) 191 208 4885; Fax. (+44) 191 208 4961.

Running Title: Environmental selection of estuarine methanotrophs.

Table SI1. Environmental conditions and lag phase (days) before the onset of methane oxidation in aerobic River Tyne sediment slurry incubations.

|  | **Environmental conditions** | | | | | | | |
| --- | --- | --- | --- | --- | --- | --- | --- | --- |
| **Temperature (°C)** | **4** | **8** | **15** | **21** | **30** | **40** | **50** | **60** |
| Lag phase (days) | 13 | 10 | 3 | 3 | 3 | 3 | 2 | N/A |
| Length of incubation (days) | 28.0 | 21.0 | 8.0 | 7.0 | 7.0 | 7.0 | 10.0 | 10.0 |
| **pH** | **4** | **5** | **6** | **7** | **8** | **9** |  |  |
| Lag phase (days) | 13 | 5 | 1 | 1 | 1 | 6 |  |  |
| Length of incubation (days) | 26.0 | 13.0 | 5.0 | 5.0 | 5.0 | 11.0 |  |  |
| **Salinity (g L^-1^) at 21°C^a^** | **1** | **15** | **35** | **70** | **120** | **150** |  |  |
| Lag phase (days) | 2 | 3.8 | 3.8 | 17 | N/A | N/A |  |  |
| Length of incubation (days) | 3.8 | 6.8 | 10.0 | 36.0 | 66.0 | 66.0 |  |  |
| **Salinity (g L^-1^) at 40°C^a^** | **1** | **15** | **35** | **70** | **120** | **150** |  |  |
| Lag phase (days) | 2 | 2 | 3.8 | 11 | N/A | N/A |  |  |
| Length of incubation (days) | 3.8 | 3.8 | 6.8 | 45.0 | 66.0 | 66.0 |  |  |
| **Methane concentration (%)** | **0.1** | **0.5** | **1** | **5** |  |  |  |  |
| Lag phase (days) | 3.4 | 2.0 | 2.0 | 2.0 |  |  |  |  |
| Length of incubation (days)^a^ | 9.2 | 6.1 | 5.0 | 5.0 |  |  |  |  |

Sediment slurries were prepared in glass serum bottles (60 ml) which comprised sterile growth medium (22 ml, Widdel & Bak, 1992), homogenized surface sediment (~3.5 g) and headspace (35 ml). Growth medium in sediment slurries was pH 7.5 ±0.7 with 7 g L^-1^ NaCl, incubation was at 21°C with 5% CH_4_ addition to the headspace, except where indicated above.
Incubation periods (days) were characterised by two phases of methanotroph activity, an initial lag phase without methane consumption and a period in which methane removal occurred which was linear and from which maximal methane oxidation rates were calculated.
^a^Based on high rates of methane oxidation observed in the temperature experiment (Fig 1), experiments subjected to different salinities were incubated at both 21°C and 40°C.
N/A - not applicable, as methane consumption was not detected in the incubations.

Table SI2. Sequence identity of methanotrophs in aerobic methane-oxidising sediment slurries incubated at different temperatures.


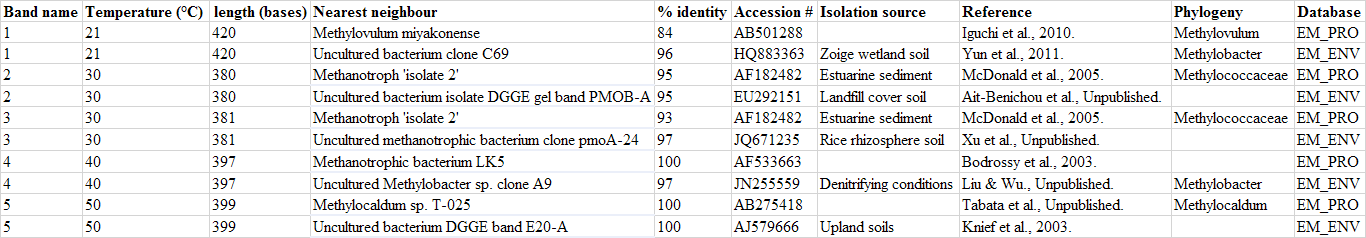


Table SI3. Sequence identity of methanotrophs in aerobic methane-oxidising sediment slurry incubations in response to methane concentration.


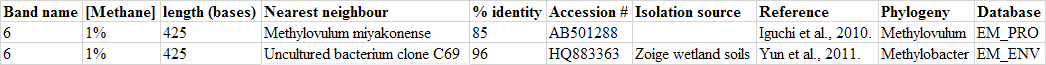


Table SI4. Sequence identity of methanotrophs in aerobic methane-oxidising sediment slurry incubations in response to pH.


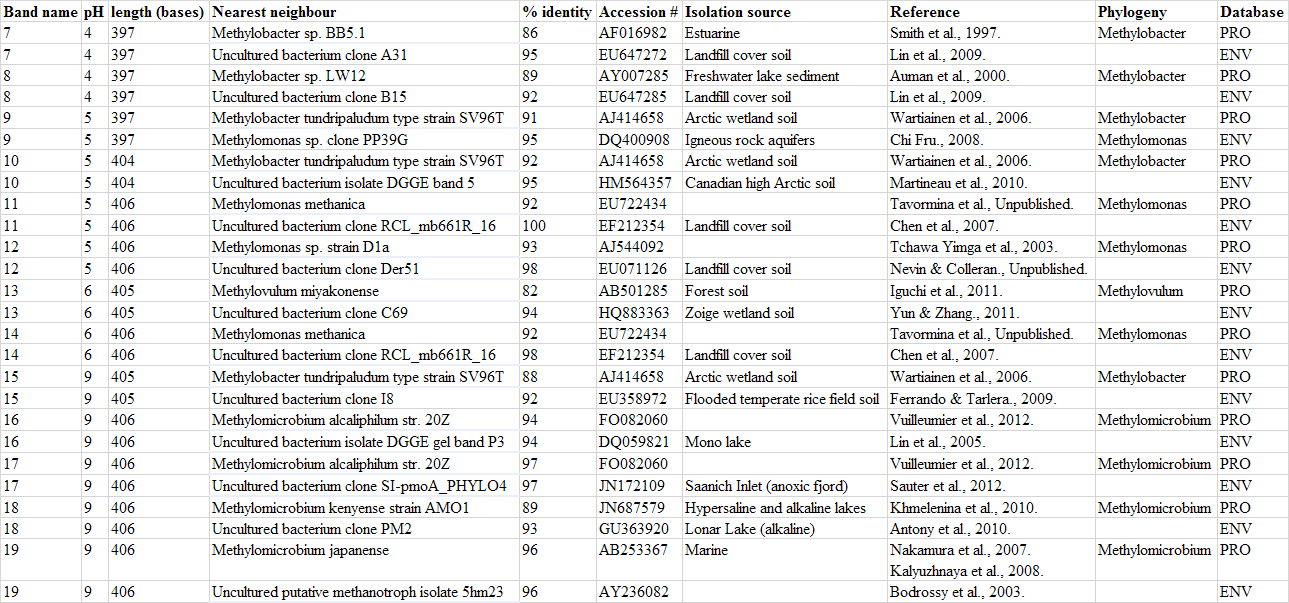


Table SI5. Sequence identity of methanotrophs in aerobic methane-oxidising sediment slurry incubations in response to salinity.


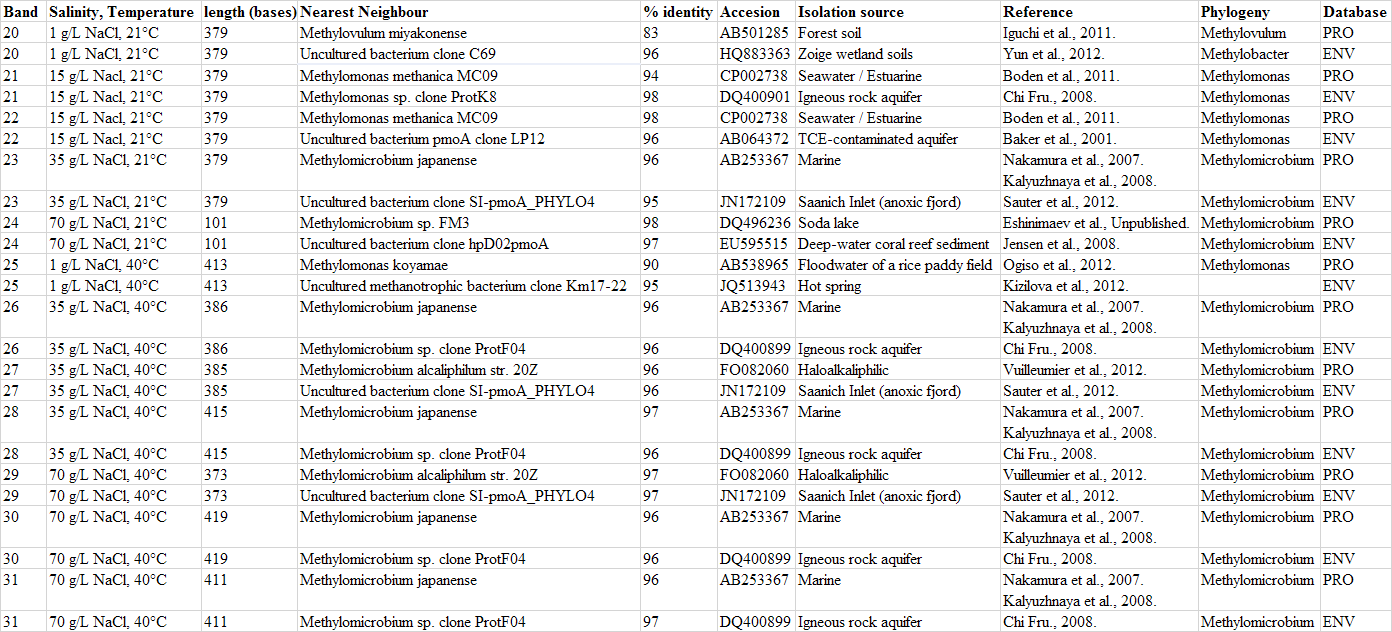


Supporting Information Table SI1. Environmental conditions and lag phase (days) before the onset of methane oxidation in aerobic River Tyne sediment slurry incubations.

Supporting Information Table SI2. Sequence identity of methanotrophs in aerobic methane-oxidising sediment slurry incubations incubated at different temperatures.

Supporting Information Table SI3. Sequence identity of methanotrophs in aerobic methane-oxidising sediment slurry incubations in response to methane concentration.

Supporting Information Table SI4. Sequence identity of methanotrophs in aerobic methane-oxidising sediment slurry incubations in response to pH.

Supporting Information Table SI5. Sequence identity of methanotrophs in aerobic methane-oxidising sediment slurry incubations in response to salinity

**Experimental Procedures**

Sediment collection

A tidally exposed surface sediment (~ 0-2 cm depth) was collected (July 2011) in 4 x sterile vessels (~650 cm^3^) at low water from the mid section (~ 13 miles from the mouth and ~ 6.5 miles below the tidal limit) of the River Tyne estuary (GPS coordinates Latitude 54°57'51.22"N, Longitude 1°40'59.38"W). Sediment was stored at 4°C prior to use.

Preparation of estuary sediment slurry incubations

Sediment slurries were prepared in glass serum bottles (60 ml, Wheaton via VWR) which comprised sterile growth medium (22 ml, Widdel & Bak, 1992), homogenized sediment (~3.5 g) and headspace (35 ml). These slurries were used to investigate methanotroph community composition in response to environmental variables (Table SI1). Due to the large number of replicated treatments required for each condition tested the schedule of experimental setup and incubation was staggered with different conditional variables prepared with separate batches of sediment and freshly prepared growth medium. Prior to use, the batches of sediment were homogenized with a sterile glass rod. The slurries comprised sulfate free growth medium at pH 7.5 ±0.7, NaCl of 7 g L^-1^ and incubated at 21°C, except where indicated in Table SI1. To adjust pH of the carbonate-buffered growth medium NaOH or HCl (1N) was used. The headspace of slurries was amended to a final concentration of 5% methane (v/v) (BOC Ltd, UK), unless otherwise indicated in Table SI1. Controls were prepared for each experiment set which did not receive methane addition and additional controls were heat-killed (autoclaving at 121°C for 20 min). Sediment slurries were prepared in triplicate for each condition and treatment (Table SI1).

Methane oxidation in sediment slurry incubations

Methane was measured periodically by removing headspace gas (0.1 ml) from each stoppered bottle, using a gas-tight syringe (SGE, Australia). Gas samples were analysed by gas chromatography with flame ionization detection (GC-FID, Carlo Elba 5160). The GC was fitted with a Chrompak Pora plot Q coated fused silica capillary column (30 m x 0.32 mm) with a hydrogen carrier gas and fixed oven (35^o^C) and injection port (250^o^C) temperatures. Methane concentrations were determined with reference to standard gas calibrations (Scientific & Technical Gases Ltd, Newcastle –under- Lyme, UK). Profiles of methane removal as a function of time were examined graphically to determine lag phase lengths before onset of a linear methane removal phase. Methane oxidation rates for individual replicate incubations were calculated from fitted slopes spanning this linear phase. Rates of methane oxidation (µmol CH_4_ day^-1^ g^-1^ wet sediment) in methane–amended incubations were statistically compared to those in the corresponding unamended, heat-killed and other methane-amended incubations using ANOVA and Tukey’s post hoc honestly significant difference (HSD) (IBM SPSS statistics, Version 19).

Molecular Microbiology

DNA extraction

Temperature, salinity and pH experiments, were destructively sampled when methane reached a concentration level of <0.5% CH_4_ in the headspace (range 0.001-0.47%). For methane concentration experiments sediment slurries were sacrificed when methane concentrations reached <0.1% CH_4_ (range 0.009-0.04%). Unamended and heat-killed, methane-amended controls were sacrificed at corresponding times. Aliquots of sediment slurry (5 ml) were removed for microbiology and the remainder of the slurry (20 ml) was frozen (-20°C) to preserve the samples for complementary lipid analysis (to be reported elsewhere). Sediment slurry (2 ml) was centrifuged (13,000 rpm, 3 min) and supernatant removed prior to DNA extraction with the Powersoil DNA isolation kit (MO-BIO via VWR, Leicestershire, UK), according to the manufacturer’s instructions.

PCR amplification

Particulate methane monoxygenase (*pmoA*) gene

Particulate methane monoxygenase (*pmoA*) genes were PCR amplified from total DNA extracted from sediment slurries with primers A189f (Holmes *et al*., 1995) and Mb661r (Costello & Lidstrom., 1999), however it should be noted that these primers do not target *Methylocella palustris* (Dedysh *et al*, 2000), *Methylocella silvestris* (Dunfield *et al*, 2003), *Methyloferula stellata* (Vorobev *et al*, 2011) or *Verrucomicrobial* methanotrophs (Dunfield *et al*., 2007, Op den Camp *et al*., 2009). For DGGE, the A189f primer was modified at the 5’ termini with a GC-clamp (Sheffield *et al*., 1989) and degenerate bases within the Mb661r reverse primer were replaced with inosine residues (Jugnia *et al*., 2009).

PCR reactions were carried out in a total volume of 50 µl (comprising primers (1µl at 10 pmol/µl), dNTPs (1µl, 10 mM), MgCl_2_ (1.5µl, 50mM), NH_4_^+^ buffer (5µl, 10X solution), Taq polymerase (0.2µl, 5U/µl Bioline, London, UK), molecular-grade water (39.3µl, Sigma-Aldrich, UK) and DNA template (1µl)) using a PCR thermal cycler (model TC512, Techne, UK) with initial denaturation (94°C, 4 min), followed by 30 cycles (94°C for 1 min, 60-50°C for 1 min (decreased by 0.33°C every cycle) and 72°C for 3 min). This was followed by 10 additional cycles at the lowest annealing temperature (50°C), with a final extension at 72°C for 5 min. PCR products were checked (size ~510 bp and purity) on 1% agarose gels.

16S rRNA genes for Type I and II methanotrophs

Methanotroph 16S rRNA genes were detected using the forward primer, U785F (Baker et al, 2003), with the Type I (targeting *Methylomonas*, *Methylobacter*, *Methylomicrobium*, and *Methylococcus*) or Type II (targeting *Methylosinus* and *Methylocystis*) methanotroph-specific reverse primer, MethT1bR and MethT2R, respectively (Wise *et al*., 1999). Primer MethT2R does not target Type II methanotrophs from the family *Beijerinckiaceae* including the genera *Methylocapsa*, *Methylocella* and *Methyloferula*. PCR reactions were prepared as above with an initial denaturation (95°C, 3 min), followed by 30 cycles of 95°C for 30 s, 49°C (Type I) or 51°C (Type II) for 30 s and 72°C for 30s with a final extension step at 72°C for 10 min. PCR products were checked for size and purity as above. The expected amplicon size was ~221 bp (Type I) and ~232 bp (Type II).

*Methylomonas methanica* S1 and *Methylosinus trichosporium* OB3b (Whittenbury *et al*, 1970) from the NCIMB culture collection (Strains #11130 and #11131 respectively, NCIMB, Aberdeen, Scotland) were resuscitated according to NCIMB guidelines. Genomic DNA was extracted from *M. methanica* and *M. trichosporium* with the UltraClean Microbial DNA Isolation Kit (MO-BIO, Leicestershire, UK) and used as positive control DNA templates for PCR with the Type I and Type II primer sets, respectively.

DGGE of *pmoA* genes

Amplified *pmoA* genes (11 µl) were loaded onto a 10% (wt vol^-1^) acrylamide gel containing a 20-55% denaturing gradient (100% denaturant consists of 7 M urea and 40% formamide). Gels were run (60°C for 16 h at 100 V) using an INGENYphorU system (Ingeny International BV, Goes, The Netherlands), stained with SYBR gold (Invitrogen, Paisley, UK) and visualized with a BioSpectrum Imaging System (UVP, Cambridge, UK). DGGE bands were excised and eluted in sterile water (100µl) at 4°C overnight. Two microliters of DNA eluate was re-amplified with A189f /Mb661r without a GC clamp using an initial denaturation of 5 min at 94°C; 30 cycles, where 1 cycle consists of 94°C for 1 min, 58°C for 1 min, and 72°C for 1 min; and a final elongation at 72°C for 30 min (Martineau *et al*., 2010). PCR amplicons were purified using ExoSAP-IT (GE Healthcare, Buckinghamshire, UK), according to the manufacturer’s instructions.

DNA sequencing of *pmoA* gene fragments

Sequencing was performed by GeneVision (Newcastle upon Tyne, UK) using an ABI Prism 3730xl DNA sequencer with the BigDye Terminator v3.1 Cycle Sequencing Kit (Applied Biosystems, Warrington, UK). Sequence data was compared to the EMBL Nucleotide Sequence Database at the European Bioinformatics Institute (EBI) using Fasta3 (Pearson and Lipman, 1988) to identify nearest neighbours.

Phylogenetic analysis of *pmoA* genes

Sequences (30, length ~289-425 bp, Table SI1-S4) were deposited in the Genbank database with accession numbers (KF958142, KF958144-KF958167, KF958169- KF958175), except Band 24 21°C 70 g L-1 which at 101 bp in length is too short for GenBank submission (GATACTTCAACTTCTGGGGATGGACATACTTCCCAGTAAACTTCGTTTTCCCATCTAACCTGATGCCAGGTGCTATCGTATTAGACGTCATCCTGATGCTT). Partial *pmoA* nucleotide sequences were aligned to their nearest neighbours using ClustalW (Higgins et al, 1994), subsequently a neighbour-joining phylogenetic tree using MEGA5 (Tamura et al, 2011) was constructed using the p-distance method (Nei and Kumar, 2000). Bootstrap analysis was performed using 1000 replicates to determine the degree of confidence in the topology of the phylogenetic tree (Felsenstein, 1985). A *pmoA* distance of 7% has previously been shown to correspond to the 3% 16S rRNA distance level (Degelmann et al, 2010), thus broadly represents differentiation at the species level.

References of Experimental Procedures

Baker, G.C., Smith, J.J., and Cowan, D.A. (2003) Review and re-analysis of domain-specific 16S primers. J Microbiol Methods **55:** 541–555.

Costello, A.M. and Lidstrom, M.E. (1999) Molecular characterization of functional and phylogenetic genes from natural populations of methanotrophs in lake sediments. Appl Environ Microbiol **65:** 5066–5074.

Dedysh, S.N., Liesack, W., Khmelenina, V.N., Suzina, N.E., Trotsenko, Y.A., Semrau, J.D. *et al*. (2000) *Methylocella palustris* gen. nov., sp. nov., a new methane-oxidizing acidophilic bacterium from peat bogs, representing a novel subtype of serine-pathway methanotrophs. Int J Syst Evol Microbiol **50:** 955–969.

Degelmann, D.M., Borken,W., Drake, H.L., and S. Kolb. **(**2010) Different atmospheric methane-oxidizing communities in European beech and Norway spruce soils. Appl Environ Microbiol **76:** 3228–3235.

Dunfield, P.F., Khmelenina, V.N., Suzina, N.E., Trotsenko, Y.A., and Dedysh. S.N. (2003) *Methylocella silvestris* sp. nov., a novel methanotrophic bacterium isolated from an acidic forest cambisol. Int J Syst Evol Microbiol **53:** 1231–1239.

Dunfield, P.F., Yuryev, A., Senin, P., Smirnova, A.V., Stott, M.B., Hou, S. *et al*. (2007) Methane oxidation by an extremely acidophilic bacterium of the phylum *Verrucomicrobia*. Nature **450:** 879-82.

Felsenstein, J. (1985) Confidence limits on phylogenies: An approach using the bootstrap. Evolution **39:** 783-791.

Higgins D., Thompson J., and Gibson T. (1994) CLUSTAL W: improving the sensitivity of progressive multiple sequence alignment through sequence weighting, position-specific gap penalties and weight matrix choice. Nucleic Acids Res **22:** 4673-4680.

Holmes, A.J., Owens, N.J.P., and Murrell, J.C. (1995) Detection of novel marine methanotrophs using phylogenetic and functional gene probes after methane enrichment. Microbiology **141:** 1947–1955.

Jugnia, L.B., Ait-Benichou, S., Fortin, N., Cabral, A. R., and Greer. C.W. (2009) Diversity and dynamics of methanotrophs within an experimental land-fill cover soil. Soil Sci Soc Am J **73:** 1479–1487.

Nei M., and Kumar S. (2000) Molecular Evolution and Phylogenetics. Oxford University Press, New York.

Op den Camp, H.J.M., Islam, T., Stott, M.B., Harhangi, H.R., Hynes, A., Schouten, S., *et al*. (2009) Environmental, genomic and taxonomic perspectives on methanotrophic *Verrucomicrobia*. Environ Microbiol Rep **1:** 293–306.

Pearson, W.R. and Lipman, D.J., (1988) Improved tools for biological sequence comparison. Proc Natl Acad Sci USA **85:** 2444-2448.

Sheffield, V.C., Cox, D.R., Lerman, L.S., and R. M. Myers. (1989) Attachment of a 40-base-pair GC-rich sequence (GC-clamp) to genomic DNA fragments by the polymerase chain reaction results in improved detection of single-base changes. Proc Natl Acad Sci USA **86:** 232–236.

Tamura, K., Peterson, D., Peterson, N., Stecher, G., Nei, M., and Kumar, S. (2011) MEGA5: molecular evolutionary genetics analysis using maximum likelihood, evolutionary distance, and maximum parsimony methods. Mol Biol Evol **28:** 2731-2739.

Vorobev, A., Baani, M., Doronina, N., Brady, A., Liesack, W., Dunfield, P., Dedysh, S. (2011) *Methyloferula stellata* gen. nov., sp. nov., an acidophilic, obligately methanotrophic bacterium that possesses only a soluble methane monooxygenase. Int J Syst Evol Microbiol **61**: 2456-2463.

Whittenbury, R., Phillips, K.C., and Wilkinson, J.F. (1970) Enrichment isolation and some properties of methane-utilizing bacteria. J Gen Microbiol **61:** 205–218.

Widdel, F. and Bak, W. (1992) Gram-negative mesophilic sulfate-reducing bacteria. In: Balows, A., Trüper, H.G., Dworkin, M., Harder, W., Schleifer, K.-H. (Eds.), The Prokaryotes, second ed., vol. 4. Springer-Verlag, New York, pp. 3352e3379.

Wise, M.G., McArthur, J.V., and Shimkets. L.J. (1999) Methanotroph diversity in landfill soil: isolation of novel type I and type II methanotrophs whose presence was suggested by culture-independent 16S ribosomal DNA analysis. Appl Environ Microbiol **65:** 4887–4897.
